# SERPINE1 drives ferroptosis in acute respiratory distress syndrome by disrupting mitochondrial NAD^+^ homeostasis and suppressing Sirt3 activity

**DOI:** 10.1016/j.redox.2026.104146

**Published:** 2026-04-15

**Authors:** Nan Gao, Wei-Jian Zhang, Yi-Xin Qian, Song Yang, Zheng-Nan Zhang, Hao-Tian Lu, Guo-Qiang Zhang

**Affiliations:** a China-Japan Friendship Hospital, Chinese Academy of Medical Sciences & Peking Union Medical College, Beijing, People's Republic of China; b Department of Emergency, China-Japan Friendship Hospital, Beijing, 100029, People's Republic of China; c Graduate School, Peking University, Beijing, 100871, People's Republic of China

**Keywords:** ARDS, SERPINE1, Ferroptosis, Sirt3, Mitochondrial NAD^+^/NADH homeostasis

## Abstract

**Background:**

Acute Respiratory Distress Syndrome (ARDS) is characterized by alveolar epithelial injury, inflammatory dysregulation, oxidative stress, and impaired repair capacity. Ferroptosis, an iron-dependent and lipid peroxidation–driven form of regulated cell death, has emerged as a pathogenic driver of ARDS; however, the upstream molecular regulators that initiate ferroptotic signaling in alveolar epithelial cells remain poorly defined. SERPINE1 (PAI-1), a mediator of inflammation, coagulation dysfunction, and epithelial injury, is frequently elevated in sepsis and ARDS, yet its mechanistic role in ferroptosis remains unknown.

**Methods:**

Transcriptomic analysis of ARDS datasets, LPS-induced mouse models, clinical serum samples, and LPS-stimulated AT2 cells were used to assess SERPINE1 expression. Gain- and loss-of-function approaches, ferroptosis assays, mitochondrial functional analyses, NAD^+^/NADH quantification and proteomics were performed to define the regulatory relationship between SERPINE1, Sirt3, and ferroptosis. TM5275 was used to evaluate therapeutic modulation of SERPINE1 in vivo and in vitro.

**Results:**

SERPINE1 was markedly upregulated in ARDS patients, ARDS mouse lungs, and LPS-treated AT2 cells, correlating with disease severity. SERPINE1 deficiency or pharmacologic inhibition significantly reduced lung injury, suppressed ferroptosis markers (ACSL4, ALOX12), and restored ferroptosis-inhibitory proteins (SLC7A11, GPX4, FTH1). Mechanistically, SERPINE1 did not directly bind Sirt3, but instead interacted with complex I subunits NDUFB10 and the NAD^+^-consuming enzyme PARP1, disrupting mitochondrial NAD^+^ homeostasis, decreasing the NAD^+^/NADH ratio, destabilizing mitochondrial membrane potential, and suppressing Sirt3 expression. These changes amplified ferroptotic signaling under inflammatory stress.

**Conclusion:**

Our findings identify SERPINE1 as a previously unrecognized upstream regulator that integrates inflammatory signaling, mitochondrial redox imbalance, and ferroptosis to drive epithelial injury in ARDS. The newly defined SERPINE1–NAD/NADH–Sirt3 axis reveals a metabolically driven mechanism of ferroptosis and suggests that targeting SERPINE1 may represent a promising therapeutic strategy to mitigate ferroptosis and ameliorate lung injury.

## Highlights

• SERPINE1 is upregulated in ARDS patients and correlated disease severity.
• SERPINE1 suppression attenuates ferroptosis, inflammation and lung injury.
• SERPINE1 disrupts mitochondrial NAD^+^/NADH balance and suppresses Sirt3 activity.
• Targeting the SERPINE1-NAD^+^/NADH-Sirt3 axis may protect against ARDS.

## 1 Introduction

Acute Respiratory Distress Syndrome (ARDS) is a life-threatening clinical syndrome characterized by disruption of the alveolar-capillary epithelial barrier, impaired alveolar fluid clearance, and dysregulated immune and oxidative responses [1]. ARDS affects approximately 10% of patients admitted to intensive care units and more than 20% of mechanically ventilated patients, with hospital mortality ranging from 35 to 45% [2]. In cases of direct lung injury (such as pneumonia or aspiration) or systemic conditions (such as non-pulmonary sepsis or TRALI), lung epithelial injury may serve as the initiating factor for ARDS and contribute to its severity [3,4]. When AT1 cells are injured, AT2 cells proliferate and differentiate to restore epithelial integrity, however, AT2 cells are inherently more vulnerable to injury than AT1 cells [5,6]. To compensate for the dysfunction of AT1 cells, AT2 cells may undergo continuous self-renewal to maintain alveolar epithelial homeostasis [[7], [8], [9]]. ARDS triggers pro-inflammatory responses and coagulation disturbances within lung tissue, leading to the depletion of surfactant produced by type II alveolar cells. This causes increased alveolar surface tension, alveolar collapse, and worsening pulmonary edema, further damaging lung tissue and increasing susceptibility to infections [10,11]. Impaired alveolar fluid clearance is closely associated with the severity of ARDS-related shock and clinical outcomes [12,13].

SERPINE1, a member of the serine protease inhibitor (serpin) superfamily, encodes plasminogen activator inhibitor-1 (PAI-1), the principal inhibitor of tissue-type and urokinase-type plasminogen activators (tPA and uPA) [14]. Elevated PAI-1 levels correlate with increased mortality in sepsis and are influenced by genetic variants such as the SERPINE1 4G/4G polymorphism [15,16]. Beyond fibrinolysis, PAI-1 is also recognized as a component of innate antiviral immunity [17]. PAI-1 exerts multiple pro-inflammatory and tissue-injuring effects. It enhances LPS-induced neutrophil activation and promotes neutrophil migration and infiltration into inflamed tissues, independent of its coagulation function [15,18,19]. PAI-1 can also aggravate mucosal injury and inflammation by blocking tPA-mediated activation of the anti-inflammatory cytokine TGF-β [20]. In the lung, PAI-1–driven epithelial–mesenchymal transition (EMT) and programmed death of alveolar epithelial cells contribute to pulmonary fibrosis [21]. Moreover, TGF-β–induced PAI-1 expression inhibits the proteolytic activity of the proprotein convertase Furin, reduces MT1-MMP activity, and modulates hematopoietic stem cell behavior [22]. PAI-1 further promotes neutrophil accumulation and tissue damage after ischemia–reperfusion by facilitating interactions between β2 integrins (Mac-1/CD11b) and endothelial adhesion molecules such as ICAM-1 [23]. These diverse functions implicate SERPINE1 as a central mediator of lung inflammation and injury. However, its role in mitochondrial dysfunction and regulated cell death pathways remains poorly defined.

Ferroptosis, an iron-dependent form of regulated cell death driven by lipid peroxidation [24], is increasingly implicated in ARDS pathogenesis [25]. Disrupted iron homeostasis, elevated lipid peroxidation, and weakened antioxidant defenses in ARDS closely mirror ferroptosis-associated molecular features. Moreover, ferroptosis amplifies inflammation, disrupts the alveolar epithelial barrier, and exacerbates immune dysregulation, reinforcing a cycle of inflammation, oxidative stress, and cell death [26]. Although ferroptosis is increasingly recognized as a pathogenic mechanism in ARDS, the upstream triggers that initiate ferroptotic signaling in alveolar epithelial cells remain largely unknown. SERPINE1 (PAI-1), a key mediator of inflammation, coagulation imbalance, epithelial injury, and fibrosis, has been proposed as a potential regulator of cell death pathways, including ferroptosis [27]. Elevated PAI-1 levels in sepsis and inflammatory lung diseases consistently associate with worse outcomes, implying a broader role in epithelial stress responses [28]. However, whether SERPINE1 serves as an upstream regulator that integrates inflammatory signaling, redox imbalance, and ferroptosis during ARDS has not been defined.

## 2 Materials and methods

### 2.1 Ethics statements

Wild-type and SIRT3^−/−^ mice were purchased from Beijing Vital River Laboratory Animal Technology Co., Ltd. (Beijing, China; certificate no. SCXK 2021-0011). All animal procedures were approved by the Animal Ethics Committee of the China-Japan Friendship Hospital of Clinical Medical Sciences (Approval No. ZRDWLL240113). The clinical study was approved by the Ethics Committee of the China-Japan Friendship Hospital (Approval No. KY2025-440-01), and written informed consent was obtained from all participants.

### 2.2 Animal model of ARDS

To establish an ARDS model, seven-to eight-week-old male C57BL/6J mice were anesthetized with isoflurane delivered at 2 L/min using an anesthesia system. Orotracheal intubation was performed under direct visualization, after which a tracheal cannula preloaded with a guiding wire was inserted into the trachea. Once correct placement was confirmed, the guide wire was removed and the cannula secured. LPS (5 mg/kg; L2637, Sigma-Aldrich, USA), derived from *Escherichia coli* O55:B5 and dissolved in 50 μL phosphate-buffered saline (PBS; G4202, Servicebio, China), was administered intratracheally. Nicotinamide mononucleotide (NMN, 500 mg/kg; CAS No. 1094-61-7; MedChemExpress, USA) was administered by intraperitoneal injection at 1 h and 12 h after surgery. Following administration, the mice were returned to their cages for postoperative recovery.

After 48 h, mice were re-anesthetized, and bronchoalveolar lavage (BAL) was performed using 1 mL sterile saline. Lungs were excised for histological analysis, while blood samples collected via cardiac puncture were used to quantify inflammatory cytokines. Each lung was divided into 5–6 sections for staining, and remaining tissues were stored at −80 °C for subsequent analyses.

### 2.3 Patient enrollment and serum sample collection

Human serum samples were collected from adult patients diagnosed with ARDS in the Department of Emergency and the Intensive Care Unit of China-Japan Friendship Hospital between March 2024 and May 2025. ARDS was diagnosed according to the Berlin definition. The inclusion criteria were as follows: (1) age ≥18 years; (2) fulfillment of the Berlin diagnostic criteria for ARDS; and (3) blood sampling performed within 48 h after ARDS diagnosis. The exclusion criteria included: (1) age <18 years; (2) pregnancy; (3) active malignancy; (4) end-stage chronic liver or kidney disease; (5) long-term immunosuppressive therapy or autoimmune disease; and (6) refusal or inability to provide informed consent.

Healthy control serum samples were obtained from age- and sex-matched volunteers undergoing routine health examinations and without evidence of acute or chronic inflammatory disease. Peripheral venous blood was collected into serum tubes, allowed to clot at room temperature, and centrifuged at 3000 rpm for 15 min. Serum samples were then aliquoted and stored at −80 °C until ELISA analysis. The demographic and clinical characteristics of the study population are provided in the Supplementary Table S1.

### 2.4 Immunofluorescence and immunohistochemistry

Paraffin-embedded lung sections were deparaffinized, rehydrated, and subjected to antigen retrieval (G1207, Servicebio, China). Endogenous peroxidase was blocked using a peroxidase blocking reagent (PV-9001, ZSGP-BIO, China) for 10 min at room temperature. Sections were then blocked with 5% normal goat serum (PV-9001, ZSGP-BIO, China) in PBS for 60 min at 23–27 °C. Primary antibodies against PAI-1 (Cat# 66261-1-Ig, 1:300, Proteintech), Sirt3 (D22A3, 1:300, Cell Signaling Technology), GPX4 (ab125066, 1:300, Abcam), and FTH1 (ab75973, 1:200, Abcam) were applied and incubated overnight at 4 °C, followed by three PBS washes.

For immunohistochemistry, sections were incubated with a signal enhancer (PV-9001, ZSGP-BIO, China) for 20 min at 37 °C, washed, and then treated with an HRP-conjugated goat anti-rabbit IgG polymer (PV-9001, ZSGP-BIO, China) for 20 min at 37 °C. After washing, DAB chromogen (G1212, Servicebio, China) was applied for 5–8 min at room temperature. Slides were rinsed, counterstained with hematoxylin (G1004, Servicebio, China), differentiated, blued, dehydrated with graded ethanol, cleared in xylene, and mounted with neutral resin (YiLi-PathoStains, China).

For immunofluorescence, Alexa Fluor 488/594–conjugated secondary antibodies (G1259, 1:400, Servicebio, China) were applied and incubated at 37 °C for 1 h in the dark, followed by DAPI nuclear staining (G1012, Servicebio, China).

### 2.5 Western blotting

Tissues or cells were lysed in PMSF-RIPA buffer (CAS No. 329-98-6, MedChemExpress, USA). Protein concentrations were measured using a BCA assay (P0012, Beyotime, China). Samples were mixed with loading buffer (P1040, Solarbio, China) and separated using SDS-PAGE (PG212, Shanghai Epizyme Biomedical Technology, China), followed by transfer to PVDF membranes (IPVH00010, Merck, Germany). Membranes were blocked in 5% non-fat milk in TBST for 1 h at room temperature.

Primary antibodies included PAI-1 (ab222754, 1:1000, Abcam), Sirt3 (D22A3, 1:1000, CST), SLC7A11 (ab275411, 1:1000, Abcam), GPX4 (ab125066, 1:1000, Abcam), FTH1 (ab75973, 1:1000, Abcam), ACSL4 (ab155282, 1:1000, Abcam), ALOX12 (ab211506, 1:1000, Abcam), PARP1 (13371-1-AP, 1:1000, Proteintech), Acetylated lysine (ab190479, 1:1000, Abcam), NDUFB10 (ab196019, 1:1000, Abcam) and GAPDH (60004-1-Ig, 1:1000, Proteintech). Membranes were incubated overnight at 4 °C, washed, and then incubated with HRP-conjugated secondary antibodies (1:5000) for 1 h at room temperature. Protein bands were visualized using an ECL substrate (SQ201, Shanghai Epizyme Biomedical Technology, China).

### 2.6 Co-immunoprecipitation (Co-IP)

According to the manufacturer's instructions, a portion of the cell lysate was incubated overnight at 4 °C with anti–PAI-1 (ab222754, Abcam), anti–PARP1 (13371-1-AP, Proteintech), anti–NDUFB10 (ab196019, Abcam), or control IgG antibodies for immunoprecipitation. Magnetic beads (73778, Cell Signaling Technology) were then added to capture the antibody–protein complexes. Samples were heated at 100 °C for 10 min to denature the bound proteins, followed by magnetic separation of the beads and elution of the immunoprecipitated material. The resulting supernatant was collected for subsequent Western blot analysis.

### 2.7 ELISA and BALF protein measurement

Whole-blood samples were centrifuged at 3000 rpm for 15 min at room temperature, and serum was collected for ELISA detection of IL-6 (E-EL-M0044, Elabscience, China) and TNF-α (E-EL-M3063, Elabscience, China). BALF was centrifuged at 1500 rpm for 10 min at 4 °C to pellet cells. Supernatants were assayed for total protein using a BCA kit (P0012, Beyotime, China)

### 2.8 Histology

Following deparaffinization and rehydration, the sections were stained with hematoxylin (G1004, Servicebio, China) for 5–10 min, rinsed with running water, differentiated in acid alcohol, and blued in ammonia. Once counterstained with eosin for 1–2 min, the sections were dehydrated through a graded ethanol series, cleared with xylene, and mounted using neutral resin. The lung injury scoring was primarily based on the criteria established by the American Thoracic Society (ATS) [29].

### 2.9 Cell culture and treatments

Human alveolar type II epithelial cells (AT2) were obtained from MeisenCTCC (Zhejiang, China) and cultured in RPMI-1640 medium (C11875500BT, Thermo Fisher Scientific, USA) supplemented with 10% fetal bovine serum (FBS) (A5256701, Thermo Fisher Scientific, USA) and 1% penicillin-streptomycin (15140148, Thermo Fisher Scientific, USA). Lentiviral vectors expressing Sirt3 shRNA, PAI-1, or negative control RNA were constructed by Genechem (Shanghai, China). AT2 cells were infected for 12 h, followed by hygromycin selection. The Sirt3 shRNA target sequence was AAGTGTTGTTGGAAGTGGAGG. For siRNA experiments, AT2 cells were transfected with PAI-1 siRNA or non-specific control RNA and selected with puromycin (A1113803, Thermo Fisher Scientific, USA). Knockdown efficiency was confirmed by RT-PCR and western blotting.

### 2.10 FerroOrange staining

According to the manufacturer's protocol, FerroOrange (MX4558, Maokang Biotechnology, China), an active fluorescent probe, specifically reacts with Fe^2+^ in live cells to irreversibly generate an orange fluorescent product. The fluorescence is not enhanced by Fe^3+^ or other divalent metal ions. Cells were incubated with FerroOrange (1 μM) at 37 °C for 30 min in the dark, followed by PBS washing and fluorescence imaging.

### 2.11 MDA and LDH release assays

Lipid peroxidation products, specifically malondialdehyde (MDA), and lactate dehydrogenase (LDH) release were measured using the Lipid Peroxidation MDA Assay Kit (M496, DOJINDO, Japan) and the LDH Assay Kit (CK12, DOJINDO, Japan), respectively, according to the manufacturer's instructions.

### 2.12 Mitochondrial membrane potential assay using JC-1

Mitochondrial membrane potential (ΔΨm) was assessed using a JC-1 assay kit (C2003S, Beyotime, China) following the manufacturer's instructions. Briefly, cells were harvested and washed twice with warm PBS, then incubated with JC-1 working solution at 37 °C for 20 min protected from light. After incubation, the cells were washed with JC-1 buffer to remove excess dye and immediately subjected to fluorescence detection. JC-1 aggregates, formed in mitochondria with high membrane potential, emitted red fluorescence, whereas JC-1 monomers, present when the membrane potential was reduced, emitted green fluorescence. The red-to-green fluorescence ratio was calculated to evaluate changes in mitochondrial depolarization. Fluorescence images were acquired using a fluorescence microscope under appropriate excitation and emission settings.

### 2.13 Measurement of NAD^+^/NADH levels

Intracellular NAD^+^ and NADH levels were quantified using an enhanced NAD^+^/NADH assay kit (S0176S, Beyotime, China) according to the manufacturer's instructions. Briefly, cells or tissue samples were lysed, and total NAD (NADt) and NADH were measured separately following selective decomposition of NAD^+^ or NADH. The NAD^+^ content was calculated by subtracting NADH from NADt. Absorbance was measured at 450 nm using a microplate reader.

### 2.14 Measurement of intracellular ATP levels

Cellular ATP levels were determined using a luciferase-based ATP bioluminescence assay kit (E-BC-F201, Elabscience, China) according to the manufacturer's protocol. Briefly, cells or tissues were lysed, and ATP content was quantified based on the luciferin–luciferase reaction. Luminescence was detected using a microplate luminometer and normalized to protein concentration.

### 2.15 Real-time qRT-PCR

Total RNA was isolated using Trizol reagent (15596018CN, Thermo Fisher Scientific, USA) according to the manufacturer's instructions. The isolated RNA was then reverse-transcribed into cDNA using the Reverse Transcription System (RK20429; ABclonal, China) as per the manufacturer's protocol. Real-time quantitative reverse transcription PCR (qRT-PCR) was performed on the resulting cDNA using the BrightCycle Universal SYBR Green qPCR Mix with UDG (RK21219; ABclonal, China) on a QuantStudio™ 3/5 Real-Time PCR System. The qRT-PCR reactions were carried out following the manufacturer's instructions. The primer sequences used for quantitative RT–PCR were as follows: SERPINE1: forward 5′-CAGAGGTGGAGAGAGCCAGA-3′; reverse 5′-GCCGTTGAAGTAGAGGGCAT-3′. SIRT3: forward 5′-TCACAACCCCAAGCCCTTTT-3′; reverse 5′-AGCAGCCGGAGAAAGTAGTG-3′. IL-1β: forward 5′-ACCTCCAGGGACAGGATATGG-3′; reverse 5′-ACACGCAGGACAGGTACAG-3′. IL-6: forward 5′-GGCAGAAAACAACCTGAACCT-3′; reverse 5′-ATTTTCACCAGGCAAGTCTCC-3′. TNF-α: forward 5′-ATCCTGGGGGACCCAATGTA-3′; reverse 5′-AAAAGAAGGCACAGAGGCCA-3′.

### 2.16 Statistical analysis

Data are presented as mean ± standard error of the mean (SEM). All statistical analyses were performed using GraphPad Prism 9 (San Diego, CA, USA) or SPSS (IBM SPSS Statistics 27.0.1, IBM, USA). Statistical significance was determined by unpaired Student's *t*-test or two-way ANOVA. Experiments were performed with at least three independent replicates. “∗” *indicates a statistically significant difference between the corresponding group (*∗p < 0.05, ∗∗p < 0.01, ∗∗∗p < 0.001, ∗∗∗∗p < 0.0001).

## 3 Results

### 3.1 SERPINE1 is significantly upregulated in ARDS and correlates with disease severity

**Fig. 1:**
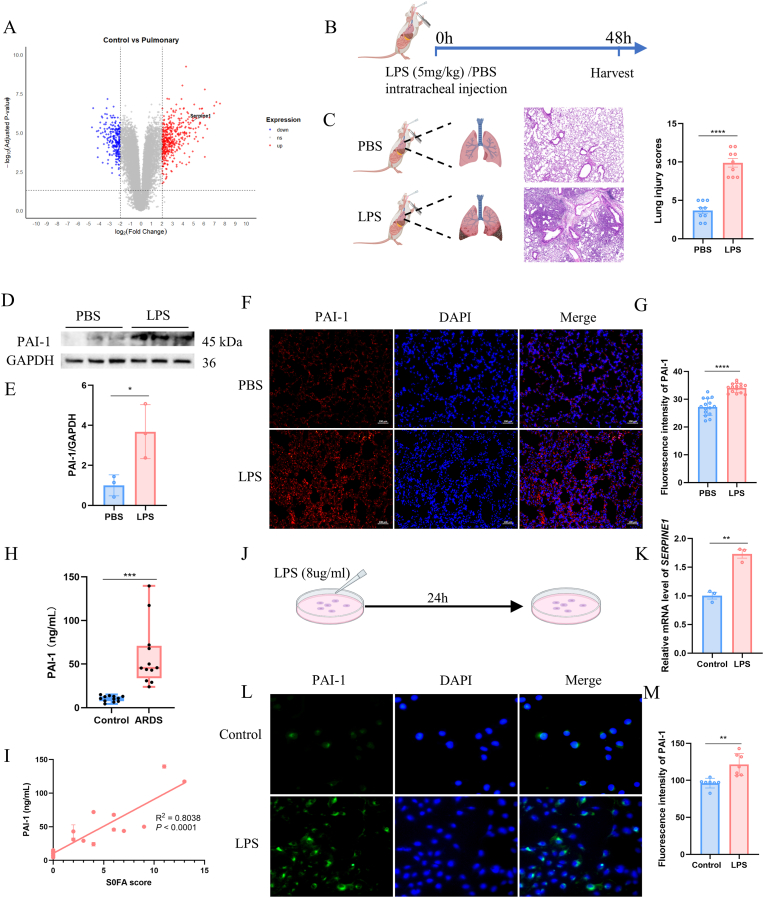
SERPINE1 is upregulated in ARDS and correlates with disease severity. (A) Volcano plot of transcriptomic data (GSE263867) showing significant upregulation of SERPINE1 in ARDS mouse lung tissues. (B) Schematic illustration of the mouse ARDS model. (C) H&E staining of lung tissues and corresponding lung injury scores. (D–E) Western blot analysis and relative quantification of PAI-1 protein levels in PBS- and LPS-treated lung tissues. (F–G) Immunofluorescence staining and quantitative analysis of PAI-1 expression in lung tissues. Scale bar = 100 μm. (H–I) Serum SERPINE1 levels in ARDS patients and healthy controls. SERPINE1 levels are significantly elevated in ARDS and positively correlate with SOFA scores (R^2^ = 0.7744, P < 0.0001). (J–K) Establishment of an in vitro ARDS model and analysis of *SERPINE1* mRNA expression in AT2 cells. (L–M) Dual immunofluorescence staining of SERPINE1 and nuclei in AT2 cells after ARDS induction, with quantitative analysis of SERPINE1 expression. Scale bar = 50 μm.

To investigate the involvement of SERPINE1 in ARDS, we analyzed publicly available transcriptomic sequencing data (GSE263867) from normal and LPS-induced ARDS mouse lung tissues. Differential expression analysis revealed a marked upregulation of SERPINE1 in ARDS tissues (Fig. 1A). We subsequently established an ARDS mouse model through intratracheal administration of LPS for 48 h. Consistently, mice subjected to intratracheal LPS administration for 48 h developed pronounced lung injury, as evidenced by extensive inflammatory cell infiltration and significantly increased lung injury scores compared with PBS-treated controls (Fig. 1B and C). Western blot analysis confirmed a substantial elevation of PAI-1 protein levels in LPS-challenged lungs (Fig. 1D and E), which was further supported by enhanced PAI-1 immunofluorescence signals within alveolar structures (Fig. 1F and G).

To determine the clinical relevance of these findings, we measured serum SERPINE1 levels in ARDS patients and healthy individuals. SERPINE1 concentrations were significantly higher in ARDS patients and showed a strong positive association with SOFA scores (R^2^ = 0.8038, P < 0.0001) (Fig. 1H–I). In vitro, LPS stimulation significantly increased SERPINE1 mRNA expression and immunofluorescence intensity in alveolar type II (AT2) cells (Fig. 1J–M). Together, these data indicate that SERPINE1 is robustly induced during ARDS and correlates with disease severity.

### 3.2 Sirt3 deficiency exacerbates ferroptosis and inflammatory lung injury in ARDS

**Fig. 2:**
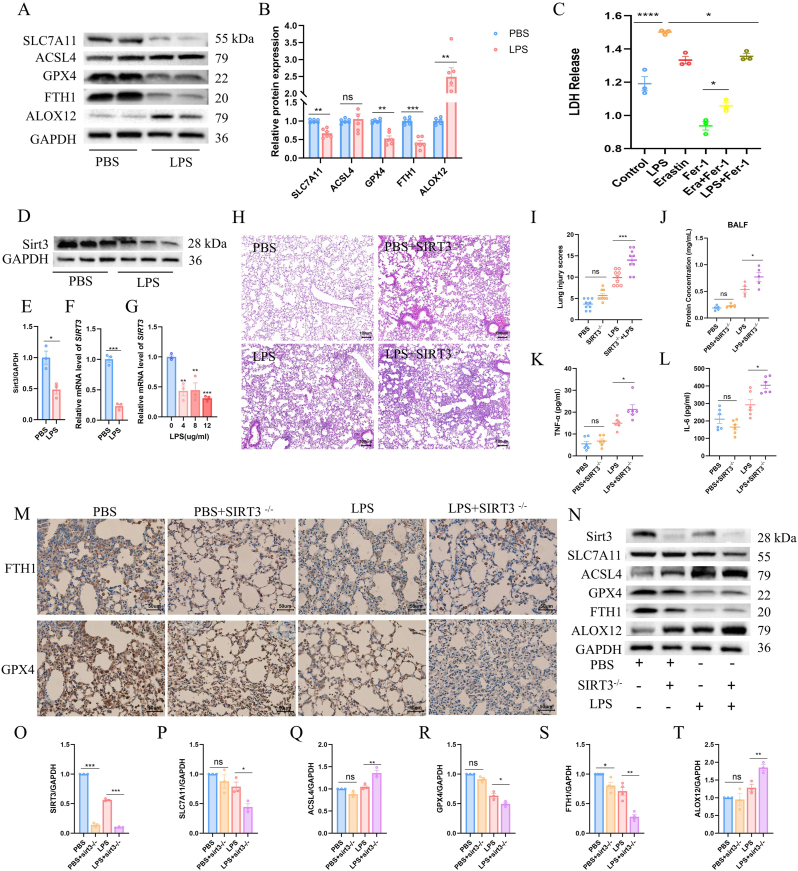
Sirt3 deficiency aggravates ferroptosis and inflammatory lung injury. (A-B) Analysis and quantification of ferroptosis-related protein expression in lung tissues from PBS and LPS groups. (C) LDH release in AT2 cells following the indicated treatments. (D–E) Western blot analysis and quantification of Sirt3 protein expression in lung tissues treated with PBS or LPS. (F) SIRT3 mRNA levels in lung tissues treated with PBS or LPS. (G) SIRT3 mRNA levels in AT2 cells exposed to increasing concentrations of LPS. (H–I) H&E staining and lung injury scores under different treatment conditions. Scale bar = 100 μm. (J) Protein concentration in bronchoalveolar lavage fluid assessed by BCA assay. (K-L) Serum TNF-α and IL-6 concentrations measured by ELISA. (M) Immunohistochemical staining of GPX4 and FTH1 in lung tissues. Scale bar = 50 μm. (N-T) Relative expression and quantification of ferroptosis-related proteins under the indicated conditions.

We next examined the contribution of ferroptosis to ARDS pathogenesis. Compared with PBS-treated mice, LPS exposure markedly increased the expression of ferroptosis-promoting proteins (ACSL4 and ALOX12) while reducing ferroptosis-inhibitory factors (SLC7A11, GPX4, and FTH1) in lung tissues (Fig. 2A and B). In AT2 cells, both LPS stimulation and the ferroptosis inducer erastin significantly increased LDH release, an effect that was effectively reversed by the ferroptosis inhibitor ferrostatin-1 (Fig. 2C).

Previous studies have established the role of Sirt3 in sepsis-induced acute lung injury. In our study, Sirt3 protein levels were significantly reduced in lung tissues from LPS-treated mice (Fig. 2D and E). Consistently, SIRT3 mRNA was also significantly decreased in the lungs of LPS-treated mice (Fig. 2F). Furthermore, SIRT3 mRNA expression was consistently downregulated in AT2 cells across increasing LPS doses (Fig. 2G). To determine the functional relevance of Sirt3, Sirt3 conditional knockout (Sirt3-CKO) mice and wild-type littermates were subjected to LPS challenge. While no differences were observed under basal conditions, LPS-treated Sirt3-CKO mice developed more severe lung injury, characterized by increased inflammatory infiltration and elevated lung injury scores (Fig. 2H and I). These mice also exhibited higher BALF protein concentrations and increased serum IL-6 and TNF-α levels compared with LPS-treated wild-type controls (Fig. 2J–L).

Consistently, immunostaining and proteomic analyses demonstrated that Sirt3 deficiency was associated with reduced expression of ferroptosis-inhibitory proteins (GPX4 and FTH1) and increased abundance of ferroptosis-promoting factors (ACSL4 and ALOX12) following LPS exposure (Fig. 2M–T). These findings identify Sirt3 as a key suppressor of ferroptosis and inflammatory lung injury in ARDS.

### 3.3 SERPINE1 suppresses Sirt3 expression and amplifies inflammatory signaling through an indirect mechanism

**Fig. 3:**
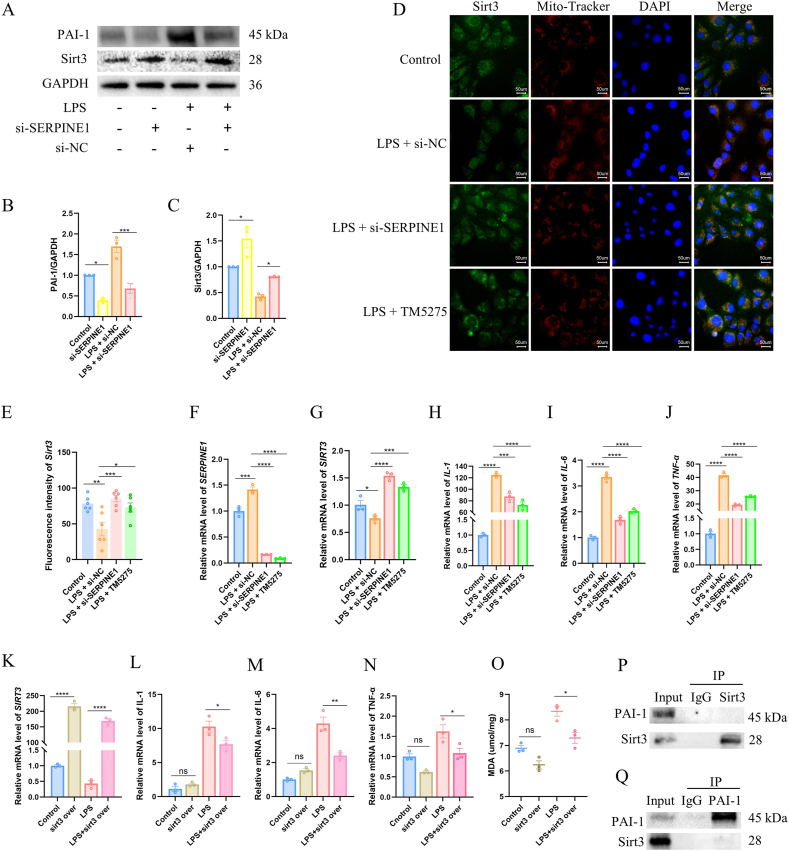
SERPINE1 suppresses Sirt3 and amplifies inflammatory responses. (A-C) Relative protein expression and quantification of Sirt3 under different treatment conditions. (D-E) Dual immunofluorescence staining of Sirt3 and mitochondria (Mito-Tracker) in AT2 cells. Green (Sirt3) and red (Mito-Tracker) signals indicate individual labeling; colocalized signals appear yellow. Scale bar = 50 μm. (F-J) Relative mRNA expression and quantification of SIRT3, IL-1β, IL-6, and TNF-α after SERPINE1 knockdown. (K–N) Relative mRNA expression and quantitative analysis in AT2 cells with Sirt3 overexpression. (O) LDH release in AT2 cells under the indicated treatments. (P-Q) Co-IP assay assessing whether endogenous PAI-1 interacts with Sirt3.

To investigate whether SERPINE1 modulates Sirt3 expression, we established SERPINE1-and Sirt3-knockdown models. Proteomic analysis demonstrated that SERPINE1 depletion consistently increased Sirt3 protein abundance, irrespective of LPS exposure (Fig. 3A–C). This finding was further validated pharmacologically using the SERPINE1 inhibitor TM5275. In control and empty-vector cells, LPS treatment markedly reduced Sirt3 fluorescence intensity, whereas SERPINE1 knockdown or TM5275 treatment significantly restored Sirt3 signal intensity under the same conditions (Fig. 3D and E).

At the transcriptional level, LPS stimulation robustly upregulated SERPINE1 mRNA expression in empty-vector cells compared with controls. Both si-SERPINE1 and TM5275 treatment significantly suppressed SERPINE1 expression relative to the LPS + si-NC group (Fig. 3F). In parallel, Sirt3 mRNA levels were significantly increased in SERPINE1-deficient and TM5275-treated cells, consistent with the observed changes at the protein level (Fig. 3G).

Functionally, under LPS stimulation, SERPINE1 knockdown and TM5275 treatment markedly reduced the expression of proinflammatory cytokines, including IL-1β, IL-6, and TNF-α (Fig. 3H–J). Given that Sirt3 deficiency has been reported to exacerbate inflammatory responses, we next examined whether Sirt3 overexpression could counteract these effects. While Sirt3 overexpression exerted minimal effects under basal conditions, it significantly attenuated LPS-induced cytokine transcription and reduced intracellular malondialdehyde (MDA) accumulation, indicating suppression of oxidative stress (Fig. 3K–O).

To assess whether SERPINE1 directly interacts with Sirt3, co-immunoprecipitation assays were performed. No detectable physical association between SERPINE1 and Sirt3 was observed (Fig. 3P and Q), suggesting that SERPINE1 regulates Sirt3 expression through an indirect, upstream mechanism rather than via direct protein–protein interaction.

### 3.4 SERPINE1 disrupts mitochondrial redox homeostasis by targeting NDUFB10 and the NAD^+^-consuming enzyme PARP1

**Fig. 4:**
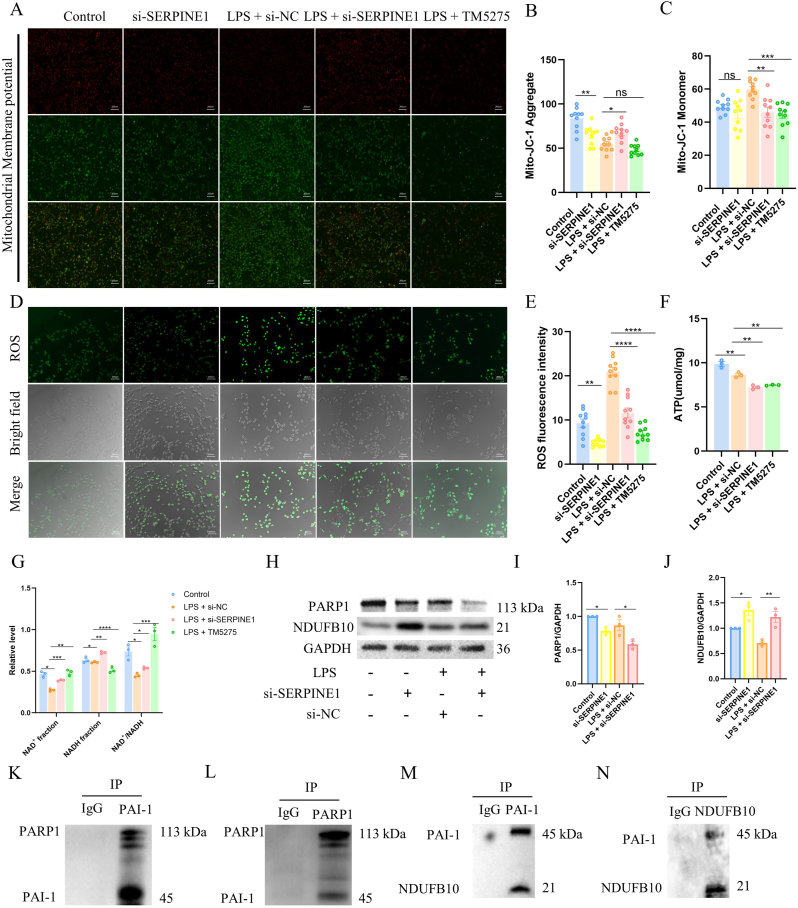
SERPINE1 modulates mitochondrial function and NAD ^+^ redox homeostasis through NDUFB10 and PARP1. (A-C) JC-1 staining of mitochondrial membrane potential. High potential results in red J-aggregates; low potential results in green monomers. (D-E) Immunofluorescence staining and quantification of intracellular ROS levels. (F) Intracellular ATP levels in AT2 cells were measured using a luminescence-based ATP assay and quantitatively analyzed. (G) Quantitative analysis of NAD^+^ fraction, NADH fraction, and the NAD^+^/NADH ratio across experimental groups. (H-J) Protein expression and relative quantification of PARP1 and NDUFB10 under different treatment conditions. (K–N) Co-IP assay to examine the interaction between endogenous PAI-1 and PARP1/NDUFB10.

To investigate how SERPINE1 regulates Sirt3, mitochondrial function was assessed using JC-1 staining. SERPINE1 depletion caused a modest reduction in red fluorescence under basal conditions, while LPS-stimulated cells showed increased red and decreased green fluorescence, indicating improved membrane potential stability. TM5275 had minimal effects (Fig. 4A–C).

We next evaluated oxidative stress. SERPINE1 deficiency reduced basal ROS levels and attenuated LPS-induced ROS accumulation, highlighting its role in mitochondrial oxidative stress (Fig. 4D and E). LPS treatment decreased ATP and NAD^+^ levels, increased NADH, and reduced the NAD^+^/NADH ratio. Both SERPINE1 knockdown and TM5275 restored NAD^+^ abundance, NAD^+^/NADH ratio, and partially improved ATP availability (Fig. 4F and G).

Given the central roles of mitochondrial complex I and the NAD^+^-consuming enzyme PARP1 in maintaining NAD redox homeostasis, we further examined their expression. At the molecular level, SERPINE1 depletion downregulated PARP1 and upregulated the complex I subunit NDUFB10 under both basal and LPS conditions (Fig. 4H–J), suggesting enhanced electron transport capacity. Co-immunoprecipitation assays demonstrated a physical interaction between SERPINE1 and PARP1/NDUFB10 (Fig. 4K–N).

These results support a model in which SERPINE1 indirectly regulates Sirt3 by targeting NDUFB10 and PARP1, thereby modulating NAD^+^/NADH homeostasis, mitochondrial membrane potential, and oxidative stress.

### 3.5 SERPINE1 modulates Sirt3-dependent ferroptosis by regulating NAD^+^ redox homeostasis

**Fig. 5:**
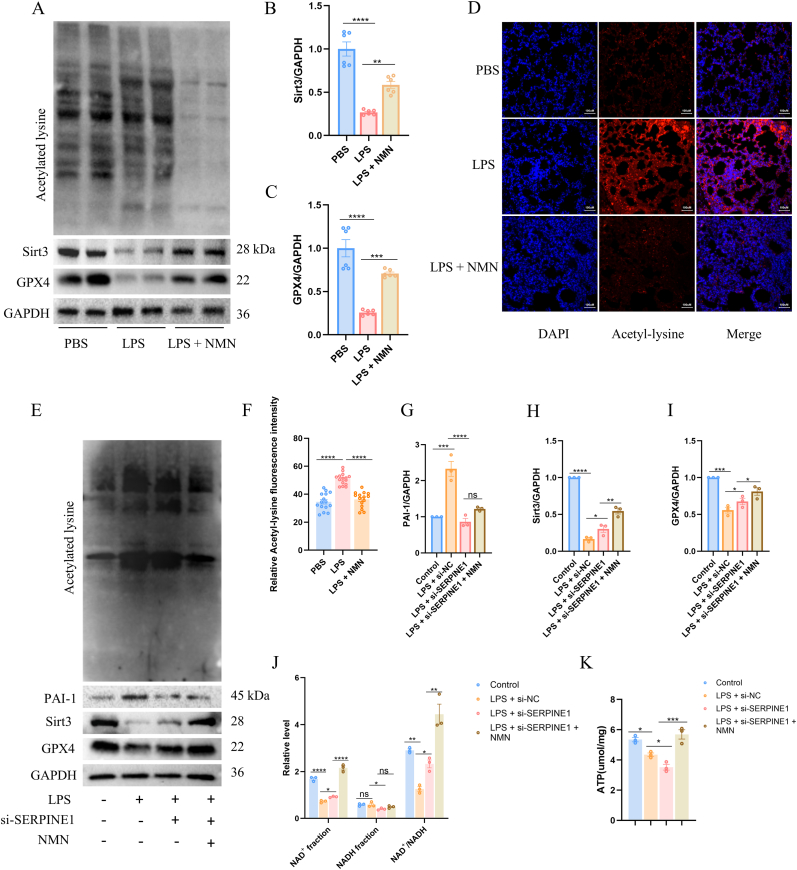
SERPINE1 regulates Sirt3 expression and ferroptosis through modulation of NAD ^+^ availability. (A–C) Western blot analysis and relative quantification of Sirt3, GPX4, and acetylated-lysine (Ac–K) under different treatment conditions. (D and F) Immunofluorescence staining and quantitative analysis of acetylated-lysine in lung tissues following NMN treatment. (E and G-I) Western blot analysis and relative quantification of PAI-1, Sirt3 and GPX4 under different treatment conditions. (J) Quantitative analysis of NAD^+^ fraction, NADH fraction, and the NAD^+^/NADH ratio across experimental groups. (K) Quantitative analysis of intracellular ATP levels in cells treated as indicated.

To determine whether SERPINE1 regulates Sirt3 expression through modulation of NAD^+^ availability, we first investigated the relationship among NAD^+^, Sirt3, and ferroptosis. Mice were administered NMN via intraperitoneal injection to elevate intracellular NAD^+^ levels, as NMN serves as a direct precursor of NAD^+^. Compared with the LPS-treated group, NMN administration markedly increased Sirt3 protein levels in lung tissues, accompanied by a corresponding upregulation of the ferroptosis-suppressive marker GPX4 (Fig. 5A–C).

Consistent with increased Sirt3 expression, the overall level of acetylated lysine in lung tissues was significantly reduced (Fig. 5A), a finding further confirmed by immunofluorescence staining (Fig. 5D and F). These results indicate that alterations in NAD^+^ availability modulate Sirt3 expression and deacetylation activity, in agreement with previous reports [30].

We next examined the relationship between SERPINE1 and NAD^+^ metabolism at the cellular level. As shown, in SERPINE1-deficient cells subjected to LPS stimulation, NMN treatment did not alter PAI-1 protein expression but further enhanced Sirt3 protein levels, concomitant with increased GPX4 expression (Fig. 5E and 5G-I).

Further analysis of intracellular NAD^+^ and NADH levels revealed that SERPINE1 knockdown significantly increased NAD^+^ abundance while reducing NADH levels, resulting in a marked elevation of the NAD^+^/NADH ratio with statistical significance (Fig. 5J). In parallel, NMN supplementation significantly increased intracellular ATP levels (Fig. 5K), indicating improved cellular energetic status.

Collectively, these findings demonstrate that SERPINE1 regulates Sirt3 expression by modulating the NAD^+^/NADH redox balance, thereby influencing ferroptosis-related molecular events. This SERPINE1–NAD^+^–Sirt3 axis represents a critical metabolic pathway linking inflammatory stress to ferroptotic regulation.

### 3.6 SERPINE1 regulates Sirt3 activity and ferroptosis through PARP1-mediated remodeling of NAD^+^ metabolism

To determine whether SERPINE1 modulates NAD^+^ homeostasis through regulation of PARP1, we treated cells with TGF-β1 and olaparib, respectively. TGF-β1 is a canonical inducer of SERPINE1, primarily driving PAI-1 transcription through a Smad-dependent signaling pathway [31,32], whereas olaparib is a well-characterized inhibitor of PARP enzymatic activity [33].

**Fig. 6:**
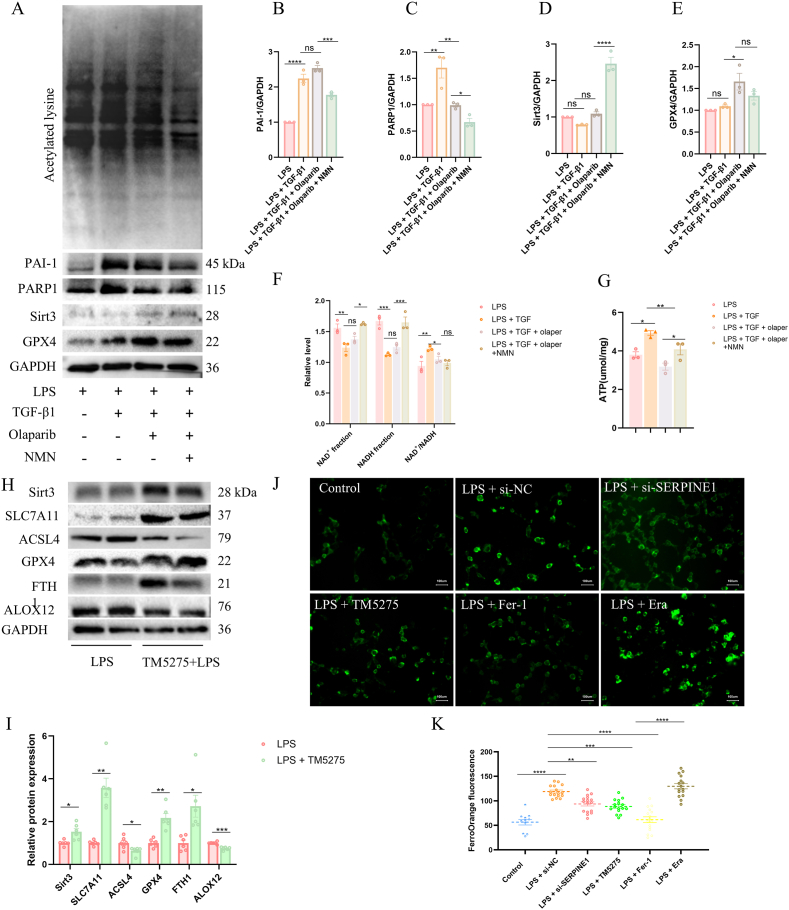
SERPINE1 regulates NAD ^+^ homeostasis and ferroptosis through PARP1-dependent mechanisms. (A–E) Western blot analysis and quantitative densitometry of PAI-1, PARP1, Sirt3, GPX4, and acetylated-lysine levels in cells treated with LPS, TGF-β1, olaparib, and NMN as indicated. (F) Quantitative analysis of NAD^+^ fraction, NADH fraction, and the NAD^+^/NADH ratio across experimental groups. (G) Quantitative analysis of intracellular ATP levels in cells treated as indicated. (H–I) Western blot analysis and relative quantification of ferroptosis-related proteins (SLC7A11, GPX4, FTH1, ACSL4, and ALOX12) in lung tissues. (J–K) Representative fluorescence images and quantitative analysis of intracellular Fe^2+^ levels under indicated conditions.

As shown, compared with the LPS-treated group, TGF-β1 stimulation markedly increased PAI-1 expression, accompanied by a significant upregulation of PARP1 protein levels. In contrast, compared with the LPS + TGF-β1 group, the addition of olaparib did not significantly alter PAI-1 expression but effectively suppressed PARP1 levels (Fig. 6A–C), and was associated with a concomitant increase in the ferroptosis-suppressive protein GPX4 (Fig. 6A and E). Notably, Sirt3 expression did not exhibit a statistically significant change following olaparib treatment relative to the LPS + TGF-β1 group (Fig. 6A and D).

In contrast, NMN supplementation markedly suppressed PARP1 expression while significantly enhancing Sirt3 protein levels (Fig. 6A and 6C–D). Consistent with increased Sirt3 activity, the overall level of acetylated lysine was correspondingly reduced (Fig. 6A), indicating enhanced deacetylation capacity.

We further examined intracellular NAD^+^ and NADH levels. Following TGF-β1 stimulation, both NAD^+^ and NADH levels were reduced; however, the NAD^+^/NADH ratio was significantly increased. Compared with the LPS + TGF-β1 group, olaparib treatment did not induce significant changes in absolute NAD^+^ or NADH levels but exhibited a trend toward reduction of the NAD^+^/NADH ratio (Fig. 6F). In parallel, TGF-β1 significantly increased intracellular ATP levels compared with LPS alone, an effect that was reversed by olaparib. In contrast, NMN supplementation markedly restored ATP levels in the presence of TGF-β1 and olaparib (Fig. 6G).

To validate the role of SERPINE1 in ferroptosis regulation in vivo, mice were administered TM5275 via oral gavage. Compared with the LPS group, lung tissues from TM5275-treated mice exhibited markedly increased expression of ferroptosis-inhibitory proteins, including SLC7A11, GPX4, and FTH1, accompanied by significant reductions in ferroptosis-promoting factors such as ACSL4 and ALOX12 (Fig. 6H and I).

Consistent with these findings, Fe^2+^ fluorescence imaging demonstrated that LPS induced a robust increase in intracellular ferrous iron levels, which was further amplified by erastin and markedly attenuated by Fer-1 treatment. Although Serpine1 deficiency partially reduced Fe^2+^ accumulation under LPS-stimulated conditions, Fe^2+^ levels remained elevated compared with those in the control group (Fig. 6J and K), indicating partial but incomplete rescue of ferroptotic dysregulation.

## 4 Discussion

In this study, we identify SERPINE1 (PAI-1) as a critical upstream regulator of ferroptosis in ARDS and reveal a previously unrecognized metabolic mechanism linking SERPINE1 to mitochondrial dysfunction. SERPINE1 expression was markedly elevated in ARDS mouse lungs, in the serum of ARDS patients, and in LPS-stimulated AT2 cells. Genetic deletion or pharmacologic inhibition of SERPINE1 significantly alleviated lung injury and suppressed ferroptosis, indicating that SERPINE1 plays a pathogenic role in ARDS progression. Mechanistically, SERPINE1 interacted with components of the NAD/NADH transport complex and its regulatory factors, disrupted mitochondrial NAD^+^ homeostasis, and subsequently inhibited the mitochondrial deacetylase Sirt3, thereby amplifying ferroptotic signaling. These findings position SERPINE1 as a molecular node integrating inflammatory stress, redox imbalance, and ferroptosis during ARDS.

Our mechanistic analyses uncover a novel axis through which SERPINE1 promotes ferroptosis. Although SERPINE1 knockdown markedly increased Sirt3 protein abundance, co-immunoprecipitation assays showed no direct binding between the two proteins. Instead, SERPINE1 interacts with NDUFB10 and PARP1, which are core components of the mitochondrial NAD/NADH transport and consumption machinery. SERPINE1 deficiency improved mitochondrial membrane potential, increased the NAD^+^/NADH ratio, enhanced NDUFB10 expression, and concurrently suppressed PARP1 expression. Because Sirt3 requires NAD^+^ as an essential cofactor to maintain mitochondrial antioxidant capacity [[34], [35], [36]], SERPINE1-mediated NAD^+^ depletion likely suppresses Sirt3 activity and sensitizes cells to ferroptosis. Thus, our findings redefine SERPINE1 as an upstream metabolic regulator of ferroptosis rather than a simple effector of inflammation.

These results expand our understanding of SERPINE1 biology and its role in ARDS pathogenesis. Previous studies primarily focused on PAI-1 as a mediator of inflammation, coagulation imbalance, and fibrosis [20,37,38]. However, its contribution to mitochondrial metabolism and regulated cell death pathways has not been thoroughly explored. Likewise, while ferroptosis has been implicated in ARDS based on evidence of iron overload, lipid peroxidation, and antioxidant depletion, the upstream drivers of epithelial ferroptosis remain incompletely understood [39]. By establishing the SERPINE1–NAD/NADH–Sirt3 axis, our study identifies PAI-1 as a modulator of mitochondrial redox balance and reveals a novel mechanism of ferroptosis induction relevant to ARDS pathogenesis.

These mechanistic insights also provide clinically relevant implications. Elevated PAI-1 levels in sepsis and ARDS patients have long been associated with poor outcomes [[40], [41], [42]], yet the underlying molecular basis has been unclear. Our data suggest that high SERPINE1 expression may directly promote epithelial ferroptosis, thereby worsening lung injury and impairing tissue repair. Treatment with the PAI-1 inhibitor TM5275 effectively reduced ferroptosis-related protein expression and decreased inflammatory cytokine production, supporting its potential therapeutic utility.

This study incorporates multi-level validation using clinical samples, in vivo models, in vitro mechanistic assays, and unbiased proteomics. Nevertheless, several limitations should be acknowledged. First, although the LPS model captures key features of ARDS, additional models (viral or aspiration-induced ARDS) are needed to generalize our findings. Second, SERPINE1 expression is regulated by multiple upstream signaling pathways, including TGF-β/Smad, inflammatory transcriptional programs such as NF-κB, and oxidative stress-associated signals [31,43,44]. These upstream mediators may themselves also influence mitochondrial dysfunction, ferroptosis, and tissue injury in ARDS [45,46]. Our present study focused primarily on identifying SERPINE1 as a functional regulator of the NAD^+^–Sirt3 axis; however, further studies are needed to delineate the upstream signaling network governing SERPINE1 induction in this context. Third, although we identified interactions between SERPINE1 and the NAD/NADH transport complex, more detailed biochemical analyses will be required to fully elucidate the dynamics of mitochondrial NAD^+^ metabolism. Fourth, the cell-type–specific relevance of the SERPINE1–Sirt3 axis, particularly within endothelial and immune cell compartments, warrants further investigation. Elucidating the broader metabolic consequences of SERPINE1 signaling may reveal additional therapeutic targets for ARDS and other inflammatory lung diseases.

## 5 Conclusions

Our findings identify SERPINE1 as a central metabolic regulator that integrates inflammatory signaling, mitochondrial redox imbalance, and ferroptosis to drive epithelial injury in ARDS. Targeting the SERPINE1–NAD/NADH–Sirt3 axis may offer a promising therapeutic strategy to mitigate ferroptosis and improve outcomes in patients with ARDS.

## CRediT authorship contribution statement

**Nan Gao:** Conceptualization, Methodology, Writing – original draft. **Wei-Jian Zhang:** Visualization, Writing – review & editing. **Yi-Xin Qian:** Investigation, Project administration. **Song Yang:** Methodology. **Zheng-Nan Zhang:** Methodology. **Hao-Tian Lu:** Supervision, Visualization. **Guo-Qiang Zhang:** Conceptualization, Funding acquisition, Writing – original draft, Writing – review & editing.

## Declaration of competing interest

The authors Declare that they have no known competing financial interests or personal relationships that could have appeared to influence that work reported in this paper.

## Data Availability

Data will be made available on request.

## Abbreviations

ARDS: acute respiratory distress syndrome
ALI: acute lung injury
AT1: alveolar type I epithelial cells
AT2: alveolar type II epithelial cells
BALF: bronchoalveolar lavage fluid
ELISA: enzyme-linked immunosorbent assay
Fe^2+^: ferrous iron
Fer-1: ferrostatin-1
GPX4: glutathione peroxidase 4
IL: interleukin
JC-1: mitochondrial membrane potential–sensitive dye
LDH: lactate dehydrogenase
LPS: lipopolysaccharide;
MDA: malondialdehyde
NMN: nicotinamide mononucleotide;
NAD^+^: nicotinamide adenine dinucleotide;
NADH: reduced nicotinamide adenine dinucleotide;
PAI-1: plasminogen activator inhibitor-1
PARP1: poly(ADP-ribose) polymerase 1
qRT-PCR: quantitative real-time polymerase chain reaction
ROS: reactive oxygen species
SERPINE1: serpin family E member 1
SIRT3 (Sirt3): sirtuin 3
SOFA: Sequential Organ Failure Assessment
TNF-α: tumor necrosis factor-α.
