# Supplementary material for: SERPINE1 drives ferroptosis in acute respiratory distress syndrome by disrupting mitochondrial NAD+ homeostasis and suppressing Sirt3 activity
Source: Redox Biol. 2026 Apr 15;94:104146. doi: 10.1016/j.redox.2026.104146 (PMC13234598; doi:10.1016/j.redox.2026.104146)
Supplement: Multimedia component 1 [file mmc1.docx]

| Variable | Healthy controls (n = 12) | ARDS patients (n = 12) | P value |
| --- | --- | --- | --- |
| Age, years | 63.75 ± 4.11 | 63.58 ± 11.56 | 0.58 |
| Sex, male/female | 8/4 | 10/2 | 0.64 |
| SOFA score | — | 5.92 ± 3.68 | — |
| PaO₂/FiO₂, mmHg | — | 166.60 ± 27.30 | — |
| Mechanical ventilation, n (%) | 0 (0%) | 5 (41.67%) | — |
| APACHE II score | — | 14.17 ± 2.29 | — |

Table S1: Demographic and clinical characteristics of healthy controls and ARDS patients.
